# Retraction: Insulin Pump Therapy vs Multiple Daily Insulin Injections for Glycemic Control in Children With Type 1 Diabetes: A Systematic Review and Meta-Analysis

**DOI:** 10.7759/cureus.r154

**Published:** 2024-12-19

**Authors:** Ernesto Calderon Martinez, Jaqueline L Castillo, Stephin Zachariah Saji, Daniel Stein, Tayyaba J Khan, Rosa F Guardado Williams, Irma D Munguía, Victor Sebastian Arruarana, Karen Velasquez

**Affiliations:** 1 Digital Health, Universidad Nacional Autónoma de México, Mexico, MEX; 2 General Medicine, Universidad Autonoma de Guadalajara, Guadalajara, MEX; 3 Graduate Medical Education, Our Lady of Fatima University, Valenzuela, PHL; 4 General Medicine, St. George's University, St. George's, GRD; 5 Medicine, Liaquat University of Medical and Health Sciences, Jamshoro, PAK; 6 General Medicine, National Autonomous University of Honduras, Tegucigalpa, HND; 7 Internal Medicine, Brookdale University Hospital Medical Center, New York, USA; 8 General Medicine, University of El Salvador, San Salvador, SLV

This article has been retracted by the Editor-in-Chief due to the presence of errors made during data calculation. The authors required the HbA1c values before and after the intervention to accurately compute the median and the standard deviation (SD). Unfortunately, there was a mistake in the calculations: Excel misinterpreted the "-" sign as the beginning of a formula instead of indicating a negative value, and it was not noticed for the R software coding. Additionally, there was an error in selecting the pre-intervention HbA1c value of one article. The authors mistakenly used the median and SD of diabetes duration instead of the HbA1c pre-intervention values due to a lack of clarity in the original article. As a result, the decision was made to retract this article.

